# Musculoskeletal Adverse Event Signals Associated With Semaglutide, Liraglutide, and Tirzepatide in the US FDA Adverse Event Reporting System

**DOI:** 10.7759/cureus.116758

**Published:** 2026-09-22

**Authors:** Akshita Wadhwa, Dhruv Patel, Andrew Bouras, Sajan Shah, Sonia Daryanani

**Affiliations:** 1 Osteopathic Medicine, Nova Southeastern University Dr. Kiran C. Patel College of Osteopathic Medicine, Clearwater, USA; 2 Internal Medicine, Nova Southeastern University Dr. Kiran C. Patel College of Osteopathic Medicine, Davie, USA

**Keywords:** adverse event reporting, glucagon-like peptide-1 receptor agonists, muscle atrophy, musculoskeletal adverse events, pharmacovigilance

## Abstract

Background: Glucagon-like peptide-1 receptor agonists (GLP-1 RAs) are widely prescribed for type 2 diabetes and obesity, yet their musculoskeletal reporting profile remains incompletely characterized. Lean-mass loss during treatment has raised concern about muscle atrophy and related complications.

Methods: In this retrospective cross-sectional pharmacovigilance study, we analyzed 20,006,989 safety reports in the Food and Drug Administration Adverse Event Reporting System (FAERS) with initial receipt dates through January 27, 2026. Semaglutide, liraglutide, tirzepatide, and six dipeptidyl peptidase-4 or sodium-glucose cotransporter-2 inhibitors were each evaluated against all other eligible FAERS reports. These were not direct comparisons between drugs. Reporting odds ratios (RORs) and proportional reporting ratios (PRRs) were calculated across 30 Medical Dictionary for Regulatory Activities preferred terms. Signals were defined using the ROR criterion, with PRR providing supporting evidence.

Results: Eight ROR-defined signals were identified among the three incretin-based therapies. Muscle atrophy showed the strongest disproportionality for semaglutide (ROR 3.96, 95% confidence interval (CI) 3.42-4.59; n = 182) and tirzepatide (ROR 2.29, 95% CI 1.96-2.67; n = 163), and both met the ROR and PRR thresholds. Additional lower-magnitude ROR signals included back pain, muscle spasms, and neck pain for semaglutide, and back pain, osteoarthritis, and neck pain for liraglutide. The muscle atrophy signal persisted in serious-report analyses for semaglutide (ROR 2.63; n = 85) and tirzepatide (ROR 2.06; n = 33). Among reports with recorded sex, female patients accounted for 1,398 of 2,187 liraglutide reports (63.9%), 2,654 of 3,853 semaglutide reports (68.9%), and 1,816 of 2,294 tirzepatide reports (79.2%).

Conclusions: Semaglutide and tirzepatide showed muscle atrophy reporting signals that persisted when the analysis was restricted to reports classified as serious. Prospective studies are needed to investigate these findings. These data cannot show whether the drugs cause muscle atrophy or how often it occurs among treated patients.

## Introduction

Glucagon-like peptide-1 receptor agonists (GLP-1 RAs) are a cornerstone of pharmacotherapy for type 2 diabetes mellitus and obesity. In the United States, GLP-1 RAs accounted for approximately 45 million dispensed prescriptions in 2023, with semaglutide exceeding 25 million [1]. Agents such as semaglutide and the dual glucose-dependent insulinotropic polypeptide (GIP)/GLP-1 RA tirzepatide produce mean weight reductions that can approach 15%-25% [2,3]. Tirzepatide is analyzed alongside selective GLP-1 RAs because it shares the incretin-based weight-loss mechanism, while its distinct pharmacology is maintained throughout. Expanding use in older adults and in patients receiving potentially myotoxic medications heightens the importance of describing musculoskeletal reporting patterns.

Weight loss with incretin-based therapies is not limited to adipose tissue. In a network meta-analysis, lean mass accounted for approximately 25% of total weight lost, with substantial variation by agent and body-composition method [4]. Reviews describe proportions ranging from roughly one quarter to approaching one half of total weight lost [4,5]. Lean-mass loss may be clinically important in patients already at risk for sarcopenia [6]. Under the revised European consensus, low muscle strength indicates probable sarcopenia, while low muscle quantity or quality confirms the diagnosis [7]. Musculoskeletal concerns also extend to bone health. In a retrospective study, 255 semaglutide or tirzepatide users were matched with 255 controls. Total-hip bone mineral density declined similarly overall, although patients without diabetes had slightly greater annualized total-hip loss than controls (-1.0% vs. -0.6%) [8].

Spontaneous-report studies provide a different perspective on these concerns. Earlier Food and Drug Administration (FDA) Adverse Event Reporting System (FAERS) studies evaluated selected endocrine and dermatologic outcomes [9]. A fracture-focused FAERS analysis found lower fracture reporting with GLP-1 RAs (adjusted reporting odds ratio (ROR) 0.44, 95% confidence interval (CI) 0.40-0.47) [10].

Recent studies have examined sarcopenia, muscle atrophy, and other musculoskeletal events with incretin-based therapies. An analysis of VigiBase, the World Health Organization global database of individual case safety reports, found no significant sarcopenia disproportionality for GLP-1 RAs as a class (ROR 1.06, 95% CI 0.86-1.29) [11]. In contrast, an updated tirzepatide FAERS analysis identified muscle atrophy among its reporting signals through the first quarter of 2025 [12]. These studies evaluated different outcomes: sarcopenia and reported muscle atrophy are not interchangeable. A broad musculoskeletal FAERS analysis covered data through the second quarter of 2024 [13]. Another FAERS study identified muscle atrophy signals for semaglutide and tirzepatide through March 2024 [14]. A separate VigiBase analysis detected a modest class-level muscle atrophy signal for GLP-1 analogues [15]. Conversely, a 2026 meta-analysis of 43 randomized trials found no increase in six prespecified musculoskeletal adverse events, although muscle atrophy was not among those outcomes [16].

The present study updates this work using a later FAERS cutoff and a selected panel of 30 terms. It also describes reporting patterns for other antidiabetic drugs and repeats the analysis in reports classified as serious.

This study aimed to identify musculoskeletal events reported disproportionately with semaglutide, liraglutide, and tirzepatide in FAERS. Reporting patterns for dipeptidyl peptidase-4 (DPP-4) and sodium-glucose cotransporter-2 (SGLT2) inhibitors were also examined to provide context.

## Materials and methods

Study design and ethics

This was a retrospective cross-sectional pharmacovigilance analysis of de-identified, publicly available adverse event reports submitted to the FAERS [17]. Because the data are de-identified and publicly available, the study did not constitute human-subject research and institutional review board approval was not required. The relevant items from the Strengthening the Reporting of Observational Studies in Epidemiology (STROBE) checklist for cross-sectional studies were followed.

Data source

We accessed FAERS through the openFDA application programming interface (API) [18]. The analysis included 20,006,989 safety reports first received by the FDA on or before January 27, 2026, using the receivedate field. The unit of analysis was a safety report returned by openFDA, not a verified unique patient or clinical event. For each drug-event pair, n (or N in the tables) denotes the number of reports naming both the drug and the event. A report could contribute to more than one pair, so counts across pairs should not be added to estimate unique patients.

openFDA supplies the latest version of each safety report. We did not perform additional case-identifier processing or detect separate submissions describing the same clinical event. Thus, retaining the latest report version does not ensure that duplicate clinical events have been removed.

We identified drugs using the harmonized generic-name field and events using the Medical Dictionary for Regulatory Activities (MedDRA) preferred-term field. A match indicates that a drug and event appear in the same report; it does not establish that the drug caused that event. Each drug was compared with all other eligible reports, including reports without a harmonized generic name. We included all reported drug roles, including suspect, interacting, and concomitant drugs. Counts came from the API total-results field rather than downloaded individual records.

Study drugs

Three incretin-based therapies were selected as the drugs of interest: the selective GLP-1 RAs semaglutide (78,284 total reports) and liraglutide (49,357 total reports) and the dual GIP/GLP-1 RA tirzepatide (120,921 total reports). Tirzepatide is not a selective GLP-1 RA; it is included because it shares the incretin-based weight-loss mechanism, and its distinct pharmacology is considered when interpreting agent-level differences. Six antidiabetic comparators from two drug classes were analyzed in parallel using the same full-FAERS reference to contextualize musculoskeletal signal profiles across drug classes: DPP-4 inhibitors (sitagliptin, 90,332 reports; saxagliptin, 1,430 reports; linagliptin, 20,523 reports) and SGLT2 inhibitors (empagliflozin, 66,534 reports; dapagliflozin, 53,555 reports; canagliflozin, 30,989 reports). These comparators were selected because they share the diabetes indication with GLP-1 RAs, providing context for interpreting whether musculoskeletal signals are specific to the incretin-based agents or common across antidiabetic therapies. Each drug was evaluated separately against all other eligible FAERS reports. The comparator findings provide descriptive context; they do not directly compare drugs or adjust for differences in treatment indication.

Musculoskeletal adverse event classification

Thirty MedDRA preferred terms, using terminology returned by openFDA at query time, were selected for analysis [19]. Twenty-nine were grouped into six clinical categories: muscle disorders (myalgia, muscle spasms, muscular weakness, muscle twitching, muscle fatigue, muscle atrophy, and rhabdomyolysis), tendon disorders (tendonitis, tendon pain, tendon disorder, and tendon rupture), bone disorders (osteoporosis, bone pain, bone density decreased, osteopenia, and pathological fracture), arthropathies (arthralgia, joint swelling, joint stiffness, arthritis, and osteoarthritis), general musculoskeletal pain (back pain, pain in extremity, musculoskeletal pain, neck pain, and musculoskeletal stiffness), and fractures (fracture, hip fracture, and spinal compression fracture). Falls were evaluated as a separate exploratory preferred term and were not aggregated with fractures because of their heterogeneous etiology.

Disproportionality analysis

Two complementary disproportionality measures were applied [20,21]. The ROR and 95% CI were computed from a two-by-two table of reports with and without the index drug and event. The ROR compares the odds of an event being named in reports containing the index drug with the corresponding odds in reports not containing that drug. An ROR of 1.0 indicates equal reporting odds. Values above 1.0 indicate higher reporting odds for the index drug. The proportional reporting ratio (PRR), its 95% CI, and the chi-squared statistic were calculated in parallel. The PRR is the proportion of all reports for the index drug that name the event divided by the same proportion among all other drugs, and is interpreted on the same scale, with the chi-squared statistic summarizing the strength of the association in the underlying two-by-two table. Both measures describe only how disproportionately an event is reported within the database; because spontaneous reporting data have no prescribing denominator, neither the ROR nor the PRR estimates incidence, absolute risk, or relative risk in treated patients. Each index drug was evaluated against all other eligible FAERS reports rather than directly against a comparator drug.

A drug-event pair was flagged as a reporting signal when at least three reports named both the drug and the event and the lower limit of the ROR 95% CI exceeded 1. Supporting PRR evidence required a PRR of at least 2, a chi-squared value of at least 4, and at least three co-reports [22]. A 0.5 continuity correction was used when a contingency-table cell was zero.

We retained unadjusted screening thresholds to identify candidate reporting patterns for subsequent evaluation, rather than to make confirmatory claims across the 270 drug-event pairs. No multiple-testing correction was applied. This choice increases the possibility of false-positive signals. PRR findings and the serious-report analysis provide supporting checks, but neither controls the multiple-testing error rate or constitutes independent replication. All findings are therefore hypothesis-generating, particularly the lower-magnitude signals.

Sensitivity and demographic analyses

A sensitivity analysis repeated the ROR screen among reports carrying the FAERS serious flag; this flag applies to the report and not necessarily to the musculoskeletal preferred term. Sex distribution was described among reports with recorded male or female sex, with unknown or missing sex excluded from percentage denominators. Age was not analyzed because age fields were inconsistently populated. Annual counts of reports containing at least one selected musculoskeletal term were summarized from 2015 through 2025. Time-to-onset analysis was not performed in this count-based API design.

## Results

Disproportionality analysis

A total of 270 drug-adverse event pair analyses were conducted across nine drugs and 30 MedDRA preferred terms. Eight signals met the unadjusted ROR screening criterion among the three incretin-based therapies (Table 1).

**Table 1:** ROR-defined musculoskeletal (MSK) signals for the incretin-based therapies in FAERS N is the number of FAERS reports naming both the index drug and the listed adverse event (drug-event co-reports). In the Signal: ROR/PRR column, the first entry states whether the ROR criterion was met (at least three co-reports and an ROR lower 95% confidence bound greater than 1), and the second states whether the PRR criterion was met (PRR of at least 2 with a chi-squared value of at least 4 and at least three co-reports). The ROR criterion defined a signal; PRR was a corroborating measure. These counts do not represent verified unique patients, and a report may contribute to more than one drug-event pair. FAERS: Food and Drug Administration Adverse Event Reporting System; ROR: reporting odds ratio; CI: confidence interval; PRR: proportional reporting ratio

Drug	Adverse event	Category	N	ROR (95% CI)	PRR	Signal: ROR/PRR
Semaglutide	Muscle atrophy	Muscle disorders	182	3.96 (3.42-4.59)	3.95	Yes/yes
Tirzepatide	Muscle atrophy	Muscle disorders	163	2.29 (1.96-2.67)	2.28	Yes/yes
Semaglutide	Back pain	General MSK pain	1,091	1.25 (1.18-1.33)	1.25	Yes/no
Semaglutide	Muscle spasms	Muscle disorders	855	1.25 (1.17-1.33)	1.24	Yes/no
Liraglutide	Back pain	General MSK pain	679	1.24 (1.15-1.33)	1.23	Yes/no
Semaglutide	Neck pain	General MSK pain	257	1.23 (1.08-1.39)	1.22	Yes/no
Liraglutide	Osteoarthritis	Arthropathies	141	1.21 (1.02-1.43)	1.20	Yes/no
Liraglutide	Neck pain	General MSK pain	157	1.19 (1.02-1.39)	1.18	Yes/no

Muscle atrophy was the strongest signal for semaglutide (ROR 3.96, 95% CI 3.42-4.59, n = 182) and tirzepatide (ROR 2.29, 95% CI 1.96-2.67, n = 163). Among the three incretin-based therapies, these were the only signals that met both the ROR and PRR thresholds. The muscle atrophy signal was not detected for liraglutide (ROR 1.07, 95% CI 0.76-1.52, n = 31).

Semaglutide additionally generated signals for back pain (ROR 1.25, 95% CI 1.18-1.33, n = 1,091), muscle spasms (ROR 1.25, 95% CI 1.17-1.33, n = 855), and neck pain (ROR 1.23, 95% CI 1.08-1.39, n = 257). Liraglutide showed signals for back pain (ROR 1.24, 95% CI 1.15-1.33, n = 679), osteoarthritis (ROR 1.21, 95% CI 1.02-1.43, n = 141), and neck pain (ROR 1.19, 95% CI 1.02-1.39, n = 157). Tirzepatide generated no additional signals beyond muscle atrophy.

Several musculoskeletal outcomes showed lower-than-expected reporting. Semaglutide had inverse RORs for fracture (0.72, 95% CI 0.67-0.78), hip fracture (0.51, 95% CI 0.40-0.66), osteoporosis (0.50, 95% CI 0.40-0.62), and decreased bone density (0.16, 95% CI 0.10-0.25). Tirzepatide showed similar inverse reporting patterns. These findings are consistent with a prior FAERS analysis [10] but should not be interpreted as evidence of skeletal protection.

Comparator drug analysis

Comparator agents also generated multiple ROR-defined musculoskeletal signals. Selected examples included osteoarthritis and rhabdomyolysis for sitagliptin; muscle twitching, muscular weakness, back pain, and rhabdomyolysis for linagliptin; muscle twitching and muscular weakness for dapagliflozin; and muscle atrophy for empagliflozin (Table 2). Because each agent was analyzed separately against the full FAERS reference, these findings are descriptive context and not head-to-head comparisons.

**Table 2:** Selected musculoskeletal signals for antidiabetic comparators N is the number of FAERS reports naming both the comparator drug and the listed adverse event (drug-event co-reports). Rows were selected to illustrate the largest comparator RORs and are not a complete listing. These counts do not represent verified unique patients, and a report may contribute to more than one drug-event pair. ROR: reporting odds ratio; CI: confidence interval; DPP-4i: dipeptidyl peptidase-4 inhibitor; SGLT2i: sodium-glucose cotransporter-2 inhibitor; FAERS: Food and Drug Administration Adverse Event Reporting System

Drug	Drug class	Adverse event	N	ROR (95% CI)
Linagliptin	DPP-4i	Muscle twitching	358	16.19 (14.57-17.99)
Linagliptin	DPP-4i	Muscular weakness	515	4.72 (4.32-5.15)
Sitagliptin	DPP-4i	Osteoarthritis	634	3.00 (2.77-3.25)
Linagliptin	DPP-4i	Back pain	627	2.80 (2.58-3.03)
Sitagliptin	DPP-4i	Rhabdomyolysis	404	2.27 (2.05-2.50)
Linagliptin	DPP-4i	Rhabdomyolysis	91	2.25 (1.83-2.76)
Sitagliptin	DPP-4i	Muscle atrophy	115	2.16 (1.80-2.59)
Dapagliflozin	SGLT2i	Muscle twitching	365	6.25 (5.63-6.93)
Dapagliflozin	SGLT2i	Muscular weakness	585	2.02 (1.86-2.19)
Empagliflozin	SGLT2i	Muscle atrophy	76	1.94 (1.55-2.42)

Sensitivity analysis: serious reports

In the serious-only analysis (11,511,314 reports), the muscle atrophy signal persisted for semaglutide (ROR 2.63, 95% CI 2.13-3.26, n = 85) and tirzepatide (ROR 2.06, 95% CI 1.47-2.89, n = 33). Tirzepatide showed additional serious signals for muscle fatigue (ROR 2.67, 95% CI 1.46-4.90, n = 10) and myalgia (ROR 1.61, 95% CI 1.43-1.82, n = 265). Semaglutide demonstrated serious signals for back pain (ROR 1.30, 95% CI 1.20-1.41, n = 608) and muscle spasms (ROR 1.31, 95% CI 1.18-1.44, n = 413); a signal for falls, analyzed as a separate exploratory preferred term rather than within the fractures category, was also observed (ROR 1.23, 95% CI 1.16-1.30, n = 1,132). For liraglutide, the back pain signal persisted in the serious-only analysis (ROR 1.24, 95% CI 1.11-1.38, n = 341), and the osteoarthritis signal strengthened (ROR 1.50, 95% CI 1.26-1.78, n = 131).

Time trends

Annual co-report counts varied markedly over time (Figure 1). Counts for semaglutide and tirzepatide increased in later years, while liraglutide counts declined after an earlier peak. These raw counts were not normalized by prescribing volume and may reflect exposure, reporting propensity, database updates, and other temporal influences.

**Figure 1:**
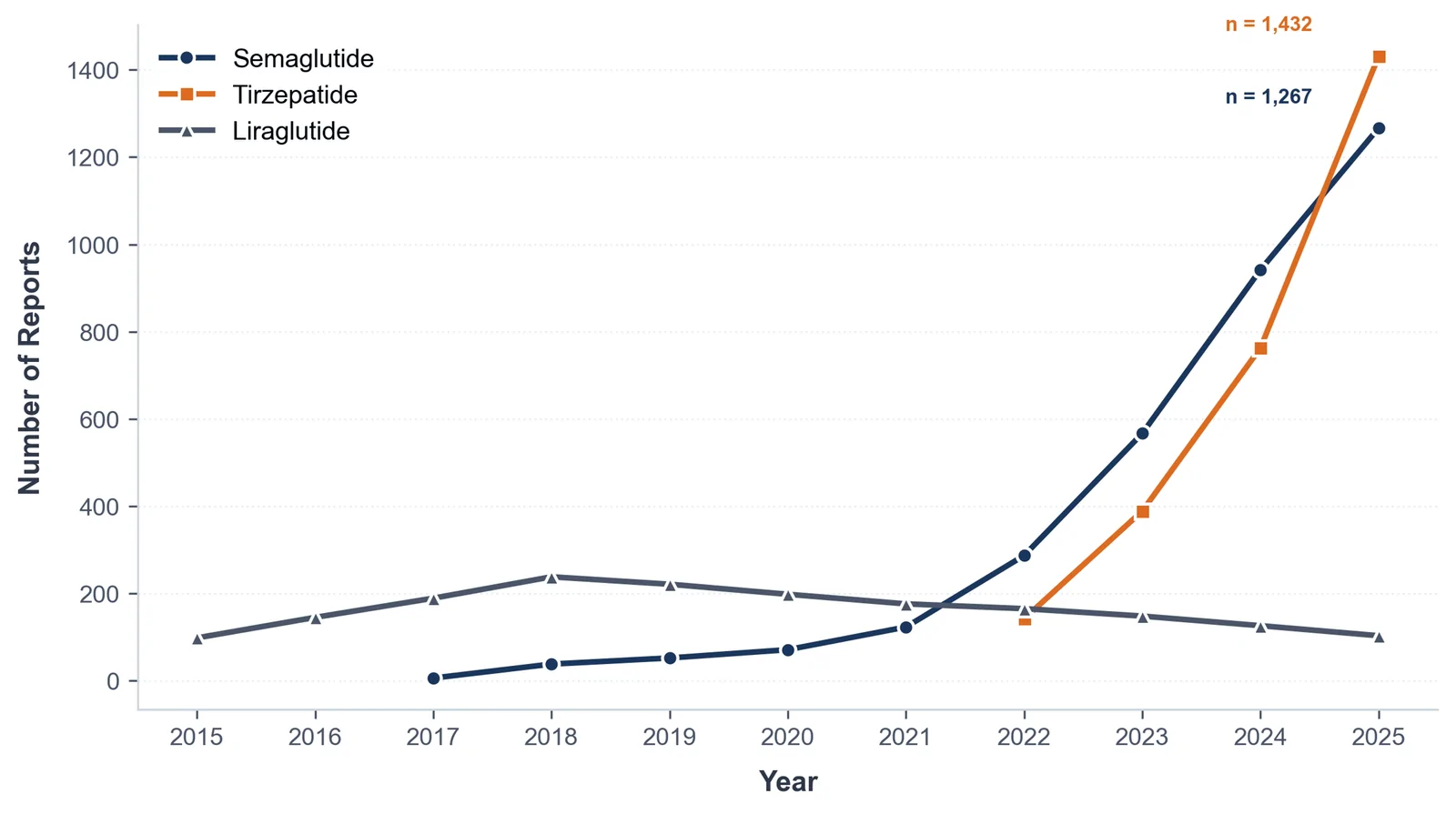
Annual musculoskeletal adverse event co-report counts for the incretin-based therapies in FAERS, 2015-2025 Raw counts were not normalized by prescribing volume. The labeled values are the 2025 counts for tirzepatide (n = 1,432) and semaglutide (n = 1,267), the final year shown. FAERS: Food and Drug Administration Adverse Event Reporting System

Sex distribution

Among reports with recorded male or female sex, female patients accounted for 2,654 of 3,853 semaglutide reports (68.9%), 1,816 of 2,294 tirzepatide reports (79.2%), and 1,398 of 2,187 liraglutide reports (63.9%) (Figure 2). Unknown or missing sex was excluded from these denominators. Sex-stratified disproportionality analysis was not performed, so these descriptive proportions should not be interpreted as sex-specific risk.

**Figure 2:**
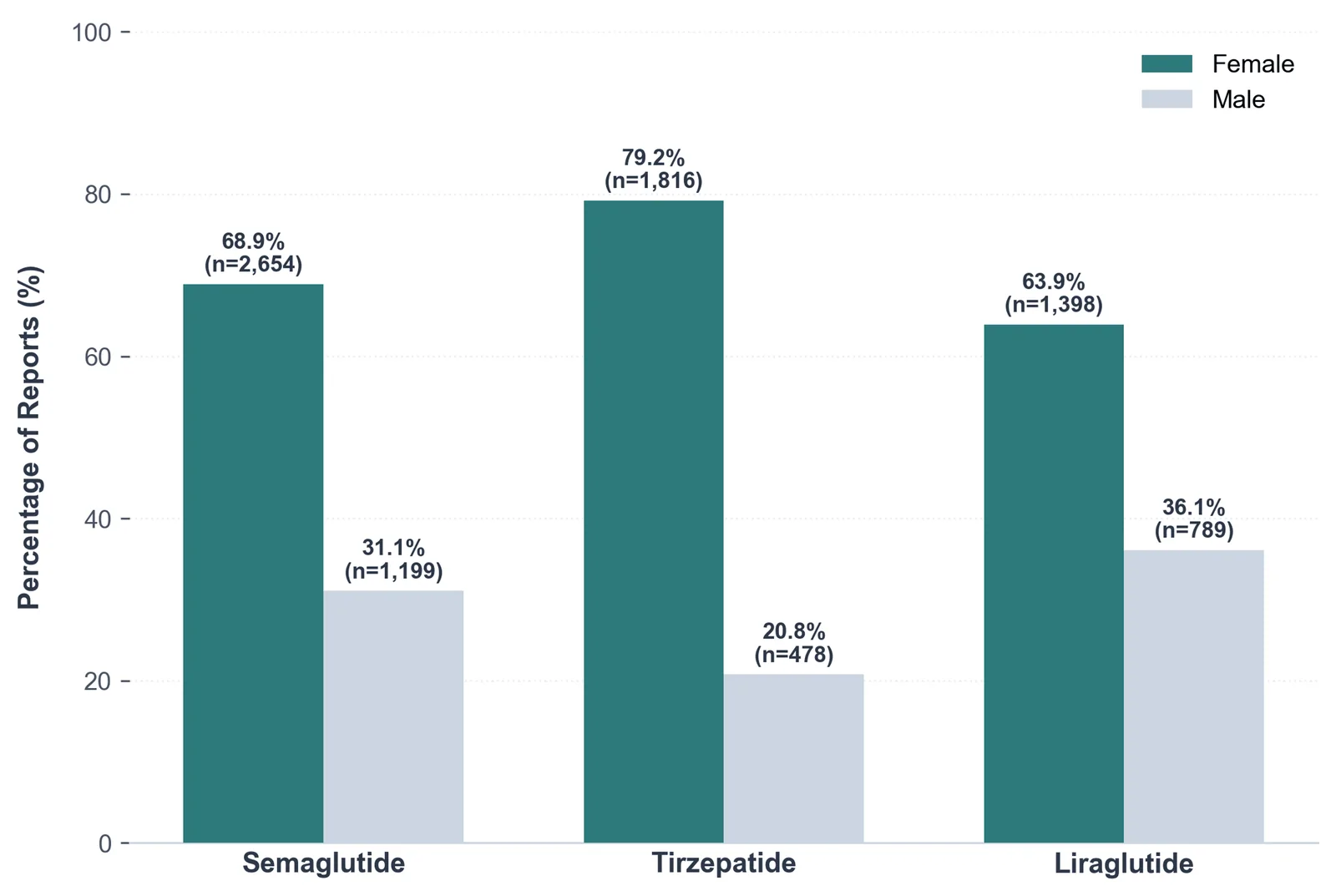
Sex distribution of musculoskeletal adverse event co-reports for the incretin-based therapies in FAERS Among reports with recorded male or female sex, female patients accounted for 1,398 of 2,187 liraglutide reports (63.9%), 2,654 of 3,853 semaglutide reports (68.9%), and 1,816 of 2,294 tirzepatide reports (79.2%). FAERS: Food and Drug Administration Adverse Event Reporting System

## Discussion

This pharmacovigilance analysis identified muscle atrophy reporting signals for semaglutide and tirzepatide. Both met the ROR and PRR criteria and persisted in the serious-report sensitivity analysis. These findings are consistent with recent FAERS studies [12-14] and a modest class-level signal in the World Health Organization global safety-report database, VigiBase [15]. The serious-report subset and earlier FAERS studies may share reports with this analysis and therefore do not establish independent replication. They are also compatible with the clinical concern that lean mass can comprise a meaningful fraction of weight lost during incretin therapy [4-6]. Liraglutide did not show a muscle atrophy signal. Although this difference may relate to its lower average weight-loss efficacy (approximately 8%) compared with semaglutide (approximately 15%) and tirzepatide (approximately 21%) [23-25], this cross-drug comparison is not a dose-response analysis and may also reflect differences in exposure, indication, reporting era, or drug role.

Preclinical studies suggest that GLP-1 RAs may affect cellular processes involved in muscle protein maintenance, energy use, and mitochondrial function. Whether these effects occur through direct receptor activation in human muscle, and whether they benefit muscle health overall, remains uncertain [5]. Caloric restriction and rapid weight loss provide additional plausible pathways for loss of lean tissue. These mechanistic hypotheses cannot be tested with spontaneous-report data.

Semaglutide produced the broadest ROR-defined profile (muscle atrophy, back pain, muscle spasms, and neck pain), tirzepatide showed only muscle atrophy in the primary screen, and liraglutide showed lower-magnitude pain and osteoarthritis signals. This heterogeneity does not establish a pharmacologic dose-response or identify a receptor-mediated mechanism. The six lower-magnitude signals did not meet the PRR criterion and are particularly susceptible to reporting bias, denominator choice, and multiplicity.

The semaglutide serious-only signal for falls (ROR 1.23, n = 1,132), analyzed as a separate exploratory preferred term outside the fractures category, is of uncertain significance. Falls have heterogeneous etiology, including orthostatic hypotension, polypharmacy, and non-musculoskeletal causes, and this signal cannot be attributed to musculoskeletal impairment. The fall signal appeared only in the serious-only sensitivity analysis and was not detected in the primary analysis, further limiting the inferences that can be drawn. The presence of both reporting signals raises a question for future study: does loss of muscle mass or function precede falls during treatment? This analysis did not establish that the two events occurred in the same patients or that one preceded the other.

Comparator agents also produced multiple ROR-defined musculoskeletal signals, including strong muscle-twitching and weakness signals for selected DPP-4 and SGLT2 inhibitors (Table 2). These results demonstrate that musculoskeletal disproportionality is not unique to incretin-based therapies. Because every agent was analyzed separately against the full FAERS reference, the estimates do not constitute head-to-head comparative safety rankings. The sitagliptin arthralgia finding is directionally consistent with the FDA warning regarding severe joint pain with DPP-4 inhibitors [26].

Fractures, hip fractures, osteoporosis, and decreased bone density were reported less often than expected for semaglutide and tirzepatide. These inverse disproportionality estimates do not demonstrate a protective effect [10,22]. Clinical data show small declines in hip bone mineral density during semaglutide or tirzepatide treatment, with slightly greater loss among patients without diabetes than matched controls [8]. The apparent difference between spontaneous fracture reporting and measured bone-density change underscores the limits of drawing clinical safety conclusions from FAERS alone.

Limitations

openFDA returns the most recent version of each safety report, but no additional record-level or cross-submission duplicate detection was performed. Reports describing the same clinical event under different identifiers may therefore remain and could bias counts and disproportionality estimates.

FAERS is a spontaneous reporting system subject to underreporting, stimulated reporting, missing data, and selective submission. It lacks prescribing denominators, so the ROR and PRR do not estimate incidence or risk. Reports without an openFDA-harmonized generic name remained in the full-database denominator, making low-magnitude estimates sensitive to reference-population specification. Inverse disproportionality should be described as lower-than-expected reporting rather than a protective drug effect. Notoriety bias [27] and the possible Weber effect [28] may also influence temporal patterns, particularly for recently introduced or highly publicized agents. The 2025 time-trend values may be incomplete because of reporting and ingestion lag.

Confounding by indication is an important limitation. Patients treated for obesity may have different baseline musculoskeletal risk than those receiving comparators primarily for diabetes, and the low-magnitude back-pain, neck-pain, and osteoarthritis signals may reflect this difference. Drug-role filtering was not performed, so co-reports could include the index drug as a concomitant rather than a suspect product. Concomitant medications, dose, duration, and treatment indication could not be reliably assessed. The separate full-FAERS analyses provide context but do not remove confounding or permit direct comparisons between drug classes.

Age was not analyzed because age fields were inconsistently populated. Reporter type and sex-stratified disproportionality were also not evaluated. Sex percentages were calculated only among reports with recorded male or female sex, and missingness limits their interpretation. These omissions are important because age, sex, reporter type, and baseline sarcopenia risk may influence both musculoskeletal events and reporting behavior.

Time-to-onset was not evaluated in this count-based API analysis, so temporality could not be established. The 270 primary drug-event comparisons were not adjusted for multiple testing, and the serious-report screen adds further comparisons. Some signals may therefore be false positives. Meeting both ROR and PRR criteria or persisting in the serious-report subset does not correct this problem, because these checks use related data. Muscle atrophy signals warrant further evaluation, but all findings remain exploratory.

Clinical implications

The muscle atrophy reporting signals for semaglutide and tirzepatide warrant further study. Relevant populations include older adults and patients with low physical activity, inadequate protein intake, chronic kidney disease, or other sarcopenia risk factors [6,7]. FAERS cannot establish that routine muscle testing before or during treatment improves outcomes. Resistance exercise and adequate protein intake are general measures to support muscle health during intentional weight loss [3]. These data do not show that such measures prevent drug-associated muscle atrophy. Prospective studies are needed to determine whether routine muscle monitoring is beneficial, which tests to use, and how often to perform them.

Future studies should use electronic health record cohorts with patients matched on relevant baseline characteristics to evaluate these reporting signals. Such studies could help assess whether the findings reflect drug effects, differences between patient groups, or reporting bias.

## Conclusions

Semaglutide and tirzepatide showed muscle atrophy reporting signals that persisted when the analysis was restricted to reports classified as serious. The other musculoskeletal findings were smaller, lacked corroboration by the PRR criterion, and should be treated as exploratory. Because FAERS cannot establish causation, incidence, temporality, or comparative safety, the findings support updated signal validation rather than changes in clinical practice. Prospective studies using objective measures of muscle mass, strength, function, treatment indication, and exposure duration are needed.
